# Integrated Biochar–Compost Amendment for *Zea mays* L. Phytoremediation in Soils Contaminated with Mining Tailings of Quiulacocha, Peru

**DOI:** 10.3390/plants14101448

**Published:** 2025-05-12

**Authors:** Paul Virú-Vasquez, Alex Pilco-Nuñez, Freddy Tineo-Cordova, César Toribio Madueño-Sulca, Teodosio Celso Quispe-Ojeda, Antonio Arroyo-Paz, Ruby Alvarez-Arteaga, Yessenia Velasquez-Zuñiga, Luis Lizardo Oscanoa-Gamarra, Juan Saldivar-Villarroel, Mary Flor Césare-Coral, Ever Nuñez-Bustamante

**Affiliations:** 1 Faculty of Environmental Engineering and Natural Resources, Universidad Nacional del Callao, Callao 07011, Peru; 2 Faculty of Chemical and Textile Engineering, Universidad Nacional de Ingeniería, Lima 15024, Peru; apilco@uni.edu.pe (A.P.-N.); ftineo@uni.edu.pe (F.T.-C.); cesar.madueno.s@uni.edu.pe (C.T.M.-S.); 3 Faculty of Environmental Engineering, Universidad Nacional de Ingeniería, Lima 15024, Peru; tquispeo@uni.edu.pe; 4 Facultad de Ingeniería, Universidad Tecnológica del Perú, Lima 15024, Peru; c25921@utp.edu.pe; 5 Facultad de Ingeniería Geológica y Metalúrgica, Universidad Nacional del Altiplano, Puno 21001, Peru; ralvarez@unap.edu.pe; 6 Facultad de Ingeniería Química, Universidad Nacional del Altiplano, Puno 21001, Peru; yessenia.velasquez@unap.edu.pe; 7 Programa de Investigación Formativa, Universidad César Vallejo, Lima 15024, Peru; loscanoaga@ucvvirtual.edu.pe; 8 Facultad de Agronomía, Universidad Nacional de Cañete, Lima 15024, Peru; jsaldivar@undc.edu.pe; 9 Faculty of Sciences, Universidad Nacional Agraria La Molina, Lima 15024, Peru; mcesare@lamolina.edu.pe; 10 Faculty of Agricultural Sciences, Universidad Nacional Autónoma de Chota, Chota 06121, Peru; enunezb@unach.edu.pe

**Keywords:** biochar, compost, mine tailings, phytoremediation, *Zea mays* L.

## Abstract

This study evaluated the phytoremediation of mine tailing-contaminated soils in Quiulacocha, Peru, using the combined application of biochar and compost, with *Zea mays L*. (maize) serving as the phytoremediator due to its high biomass production and stress tolerance. A factorial experimental design was implemented, varying two main factors: the mining tailings dose (30% and 60% *w*/*w*) and the biochar pyrolysis temperature (300 °C and 500 °C). The mine tailings were characterized by high concentrations of heavy metals and unfavourable physico-chemical properties (pH, low organic matter), whereas the biochar, produced from pine forest residues, and the compost, derived from urban organic waste, exhibited attributes that enhance soil quality. During the pot experiment, response variables including the Bioconcentration Factor (BCF) and Translocation Factor (TF) for various metals were evaluated to assess the capacity for contaminant immobilization and their distribution between plant roots and aerial tissues. The results demonstrated that the incorporation of biochar and compost significantly improved soil quality by increasing pH, cation exchange capacity, and nutrient retention, while simultaneously reducing the bioavailability of heavy metals and limiting their translocation to the aerial parts of maize. Factorial analysis further indicated that both the tailings dose and biochar pyrolysis temperature significantly influenced the efficacy of the phytoremediation process. In conclusion, the combined application of biochar and compost presents an effective and sustainable strategy for rehabilitating mine tailing-contaminated soils by stabilizing heavy metals and promoting the safe growth of *Zea mays* L.

## 1. Introduction

Large-scale mining generates massive volumes of mine tailings (fine waste from mineral processing), estimated at over 14 billion tons annually worldwide [1], generating pollution, specifically affecting the soil in many countries worldwide, including China [2], Australia [3], Spain [4], South Africa [5], Mexico [6], and Peru [7,8]. In Latin America, numerous Mining Environmental Liabilities (MELs) have been documented, notably in countries such as Ecuador [9], Peru [10], Colombia [11], and Chile [12]. MELs often result from abandoned or inadequately managed mining operations, posing ongoing environmental and health risks to surrounding communities. Addressing these liabilities is crucial for sustainable development and the protection of ecosystems in the region. These wastes contain high concentrations of toxic heavy metals, such as cadmium (Cd), lead (Pb), zinc (Zn), copper (Cu), chromium (Cr), nickel (Ni), and arsenic (As), among others [13].

Improper disposal of mine tailings enables metal-rich particles to migrate kilometres from the source via erosion and leaching, polluting soils and water bodies [14]. Heavy-metal enrichment disrupts soil biota, suppressing beneficial microbes, harming fauna, and destabilizing ecosystem functions [15], while the persistence and bioaccumulative nature of many metals permits their entry into food webs, creating chronic human health risks [16]. Remediation is particularly challenging because tailing-impacted soils are acidic, compact, nutrient-poor, and low in organic matter, conditions hostile to plant establishment [17]. Organic amendments have therefore been advocated to upgrade soil quality and attenuate in situ metal toxicity [18]. Compost supplies stabilized organic matter and nutrients that boost microbial activity and improve soil structure, aeration, and water retention, thus fostering vegetation recovery [19,20]. Biochar, a porous, carbon-rich pyrolysate, adds a high surface area, functional groups, and alkalinity, allowing it to adsorb and immobilize metals while conditioning the soil’s physical, chemical, and biological attributes [21,22,23]. Recent work shows that combining compost and biochar magnifies these individual benefits [24,25,26]: corn-straw biochar in sludge compost cut metals and antibiotic-resistance genes by up to 97.9% [27]; co-composting plant residues with biochar accelerated humification and generated a high-quality peat substitute [28]; biochar–compost blends in pesticide-laden soils under freeze–thaw cycles enhanced degradation and soil multifunctionality [29]; and earthworm-assisted sludge composting with biochar shifted cadmium into less mobile fractions and lowered associated resistance genes [30].

Phytoremediation harnesses plants to rehabilitate contaminated soils in situ, offering a low-cost and environmentally benign alternative to physicochemical methods [31]. For heavy-metal pollution, it operates chiefly through two distinct, yet complementary, mechanisms. Phytoextraction involves (i) the root uptake of soluble or weakly sorbed metal ions, (ii) xylem-mediated translocation, and (iii) sequestration in harvestable above-ground biomass; repeated cropping and biomass removal gradually deplete the contaminant pool [32]. Phytostabilization, by contrast, immobilizes metals in the root zone: root exudates and rhizosphere microbes raise pH, supply organic ligands, or promote the formation of carbonate, phosphate, and sulphide precipitates, while biochar- or compost-induced surface functional groups adsorb complex cations; the resulting reduction in metal solubility and dust generation curtails their ecological and human exposure [33]. Successful application demands species that grow rapidly, generate ample biomass, and tolerate metal stress [34]. The *Zea mays* L. satisfies the key attributes for metal phytoremediation high biomass, agronomic familiarity, broad climatic adaptability, and proven metal tolerance and has already been deployed both for phytoextraction [35] and phytostabilization [36]. Recent evidence reinforces its suitability: Atta et al. showed maize cultivars removing up to 38% Cr and 24% Pb while sustaining growth under 300 ppm stress [37]; Ahmad [38] demonstrated that P-loaded biochar co-applied with maize cut the exchangeable Cd, Pb, Cu, and Zn fractions and boosted shoot biomass two to three fold; Rosas-Castor [39] reviewed the capacity of the plants to take up and translocate arsenic, highlighting its importance where As-laden irrigation water is common. Field and pot studies in tropical soils echo these findings: biochar-amended maize improved height and dry matter while lowering soil Cu/Zn availability after 22 years of industrial contamination, and lysimeter trials recorded meaningful Cd and Zn extraction without growth penalties. Maize has also been classified as a Cr (VI) hyper accumulator under chelate-assisted conditions, with shoot concentrations rising tenfold over controls, and comprehensive reviews position it among the most promising high biomass candidates for large-scale, low-cost phytoremediation programmers. Collectively, these studies confirm that maize not only tolerates but actively removes a broad spectrum of priority metals while retaining the practical advantages of seed availability, local acceptance, and established agronomy that make it logistically attractive for remediation projects [40,41].

Growing evidence indicates that amending contaminated soils with biochar [1], compost [42] or, most effectively, their combined application can enhance phytoremediation by improving soil physical structure, increasing cation exchange capacity, and supplying essential nutrients [43]. Biochar–compost blends have successfully reduced the bioavailability of Cd and Zn [44,45] and stabilized Pb and As [46], while *Zea mays* L. has been widely investigated as a phytoextractor of Cu [47], Zn [47,48], Pb [49,50,51], and Cr [52]. In tropical field trials, integrated biochar–compost strategies improved soil water retention, boosted cation exchange capacity, and raised maize grain yields by 10–29% [24,25,26,27]. Complementary pot experiments confirmed that the co-application of the two amendments alleviates Cr phytotoxicity in maize by enhancing morphophysiological and biochemical traits [53], lowers the bioavailability and plant uptake of Cr, Ni, Pb, and Zn while stimulating biomass production, and, when combined with maize straw, modifies root exudation patterns in ways that further enrich soil fertility and yield [54]. Additional studies have shown that agro-industrial biochar–compost mixtures increase P availability and organic matter content in acidic soils, thereby optimizing maize performance [55], and that integrating these organic amendments with manures mitigates Ni toxicity, sustains photosynthesis, and promotes overall plant efficiency [56]. Nonetheless, maize-based phytoremediation is not without its caveats: several reports document appreciable Cd, Pb, and Zn transfer to kernels and fodder, raising food safety concerns; consequently, harvested biomass should be channelled to non-edible uses (e.g., bioenergy or fibre) and grain metal concentrations must be routinely monitored to ensure regulatory compliance [57].

Peru’s Quiulacocha mining tailings (QMT) deposit, created in 1930 by the Copper Corporation and now classified as an MEL unchecked disposal of pyrite-rich tailings (~50% pyrite) around an acid lagoon, has long harmed local ecosystems and nearby communities [58,59,60]. Recent climate data underline the urgency of intervention: extraordinary storms in 2021 dropped >10 mm day^−1^ of rain, shrinking the tailings’ freeboard to just 55 cm [61]; in 2022, 13.2 mm day^−1^ rainfall reduced it further to 61 cm; and intense 2023 downpours in districts such as Paucartambo confirm a continuing trend [62]. These events heighten the risk that rivers bordering the site—the Ragra and San Juan—along with adjacent soils will become conduits for heavy-metal dispersion, threatening environmental and human health through contaminated water, food chains, and direct soil contact. The magnitude of contamination caused by the Quiulacocha mine tailings in Junín, Peru, necessitates comprehensive, effective, and sustainable environmental remediation strategies. In this context, the combination of organic amendments with phytoremediation using *Zea mays* L. emerges as a promising solution for rehabilitating soils with high levels of heavy metals.

Therefore, this study focuses on evaluating the effectiveness of using biochar, compost, and *Zea mays* L. in the remediation of soil contaminated with mine tailings from Quiulacocha, providing valuable insights for the treatment of mining environmental liabilities and the mitigation of their ecosystem impacts.

## 2. Results and Discussion

### 2.1. Mine Tailing Physicochemical Characterization

The Quiulacocha mine tailings in Peru cover approximately 114 hectares and consist of roughly 79 Mt of tailings, containing about 50% pyrite by weight [63]. Mineralogical surveys report that the solid phase is dominated by sulphide minerals, principally pyrite (FeS_2_, 50–60 wt%) with accessory pyrrhotite and marcasite which oxidize to generate acid mine drainage [64]. Consistent with this assemblage, bulk chemistry shows very high total concentrations of Zn (9091 mg kg^−1^), Pb (3984 mg kg^−1^), and As (1015 mg kg^−1^) (Table 1), while the matrix remains poorly buffered (CaCO_3_ 0.35%) and low in organic matter (3.02%), leading to an acidic pH of 5.63 and elevated electrical conductivity (7.14 mS cm^−1^). These mineralogical and geochemical features confirm the high acid-generating potential and metal mobility of the deposit, underpinning the rationale for testing alkaline, functional-group-rich biochar–compost amendments to raise pH and immobilize metals in situ. For the physicochemical characterization of mining tailings that is shown, the content of organic matter (OM) is important since it can enhance metal adsorption in a tailing’s plant–soil environment [65]. The pH was acidic, and this could be due to the tailings containing more acidic minerals instead of minerals with a neutralization capacity (carbonates and hydroxides) [66]. Furthermore, if the mine tailings exhibit acidity, this will lead to a reduction in the ability of the soil to exchange metal cations and an elevation in metal solubility [67]. Also, the content of P (ppm), K (ppm), CaCO_3_ (%), and EC is shown.

### 2.2. Biochar and Compost Characterization

While this study used pine residues exclusively, feedstock composition can strongly modulate the immobilization capacity of biochar. Lignocellulosic woods such as pine typically yield chars with low ash, moderate pH, and abundant oxygenated surface groups that favour complexation and electrostatic attraction of cationic metals; agricultural straws or manures, by contrast, produce biochars richer in mineral ash (Ca, Mg, P) that promote precipitation or co-precipitation mechanisms, often raising the pH beyond 9 [68]. We therefore selected pine to maximize functional-group-driven complexation and to avoid the very high electrical conductivity associated with manure biochars, which can impair seedling emergence in tailings. Future work will incorporate a side-by-side comparison of pine, crop-straw, and poultry-litter biochars produced at 500 °C to disentangle temperature and feedstock effects. The results of the characterization of biochar and compost are shown in Table 2.

For pine biochar, as the temperature increased, the concentrations of carbon (C%), nitrogen (N%), and sulphur (S%) increased, while hydrogen (H%) and oxygen (O%) decreased. The physicochemical parameters are strongly influenced by the composition of the raw material, yet the variation in specific parameters generally follows a predictable trend with temperature. For example, as the pyrolysis temperature increases, the pH and surface area (m^2^/g) tend to rise, while the yield (%), hydrogen-to-carbon (H/C) ratio, and oxygen-to-carbon (O/C) ratio decrease [69]. Several pine-based studies indicate that the BET surface area rises from 300 °C to 500 °C (≈2.9–175.4 m^2^ g⁻^1^) but levels off—or even declines above 600 °C—due to sintering and higher ash content, while the functional groups that complex metals are progressively lost [70]. We therefore selected 500 °C in this study as the optimum compromise between specific surface area and chemical functionality.

The decrease in cation exchange capacity (CEC) observed in PB500 is attributed to the reduction in total organic matter (TOM) (39.940), as higher temperatures promote greater decomposition of organic material in the biomass. The lower CEC observed after pyrolysis at higher temperatures is also associated with a lower O/C ratio [71]. This is because higher temperatures lead to a decline in the abundance of functional groups, particularly oxygenated functional groups on the biochar surface [72]. The temperature-induced decrease in oxygen-containing surface functional groups may explain these findings [69]. Regarding pH, several studies have reported that increasing pyrolysis temperature leads to a rise in pH [72], which was also observed in this study, where the biochar exhibited a basic pH, like the findings of Cooper [73]. For electrical conductivity (EC), its value depends on the biomass type, but it generally increases with higher temperatures [74]. In this study, EC was 169.43 for PB300 and 255.60 for PB500. This increase may be attributed to higher ion mobility, reduced internal resistances, and increased thermal energy at elevated temperatures. The findings above illustrate the physicochemical properties of the compost, which exhibited a moderately alkaline pH (pH = 8.53) due to its composition. The low EC values in the following table are likely due to the minimal presence of soluble salts, which facilitates the composting process. The TOM content in the compost was lower compared to PBC300 and PBC500. Regarding the carbon-to-nitrogen (C/N) ratio, its value was lower (11.68:1), whereas it is considered optimal within the range of 25:1 to 30:1 [75]. A high C/N ratio leads to a decrease in biological activity, whereas a low C/N ratio results in nitrogen loss in the form of ammonia [76].

In Table 3, the heavy metal content in PBC300, PBC500, and compost was compared against various international regulations, including IBI [77], EBC [78], Germany (G), and Austria (AU) [79] for biochar, as well as standards from Korea, the EU, and the USA [80] for compost. Among these regulatory frameworks, the EBC and G standards appeared to be the most restrictive for heavy metal limits, particularly for cadmium (Cd) and lead (Pb), with allowable concentrations set at ≤1.5 mg/kg and ≤150 mg/kg, respectively. Comparatively, AU and IBI standards were slightly more lenient, allowing higher concentrations for some metals. For PBC300 and PBC500, heavy metal levels were generally below the strictest thresholds, with cadmium and chromium being nearly undetectable, though arsenic (As) levels were relatively high, especially in PBC500 (24.453 mg/kg). The compost, derived from municipal waste in this research, exhibited higher concentrations of heavy metals such as Cu (54.94 mg/kg) and Zn (173.63 mg/kg), although within acceptable limits under USA and EU regulations. These findings suggest that while biochar from pine biomass adheres to most international biochar standards, compost quality may require stricter monitoring to comply with the most restrictive guidelines.

Figure S2 now not only identifies the principal FTIR bands but links them explicitly to the chemisorption and precipitation routes that account for the metal stabilization observed in Table S1 (Supplementary Materials). Broad bands at 3500–3250 cm⁻^1^ (labelled O–H/N–H) originate from hydroxyl and amine groups whose lone-pair electrons donate empty d-orbitals of soft metals such as Cd^2^⁺ and Pb^2^⁺, forming inner-sphere surface complexes [81]. The shoulder at 2500–2000 cm^−1^ (C≡C/C≡O) reflects π-bond-rich alkynes and residual carbonyls that supply delocalized electrons for cation–π interactions, reinforcing Pb^2^⁺ sorption at circum-neutral pH [82]. The distinct peak near 1750 cm⁻^1^ (C=O) corresponds to carboxyl/ester carbonyls; deprotonation of –COOH groups above pH 5 generates negatively charged sites that electrostatically attract Zn^2^⁺ and Ni^2^⁺ or chelate them as bidentate complexes [83]. The envelope at 1605–1660 cm^−1^ (C=C/-CO-NH-) represents conjugated aromatics and amide linkages; the aromatic π-system provides additional cation–π adsorption, while amide carbonyls can coordinate Cu^2^⁺ via oxygen and nitrogen donors [84]. The smaller band at ≈1500 cm⁻^1^ (C=N) signals pyrrolic/hetero-aromatic N, whose basic lone pairs form strong coordination bonds with borderline metals (Cr^3^⁺, Cu^2^⁺) and contribute to Lewis-base sites on the char surface [85].

Comparison of spectra shows that PBC500 retains fewer O–H/N–H and more condensed C=C/C=N structures than PBC300, indicating a shift from ligand-rich acidic sites toward π-electron-rich aromatic domains as pyrolysis rises from 300 °C to 500 °C; this transition favours inner-sphere complexation and cation–π interactions over simple ion exchange, explaining the superior immobilization of Pb, As, and Cd recorded for the 500 °C biochar treatments. Compost augments this effect by supplying additional –COOH/phenolic-OH groups and raising soil pH, thereby promoting precipitation of metals as carbonates or phosphates and further reducing their bioavailability.

### 2.3. BCF and TF Results

The metal concentration results in the soil treatments (Table S1) show that arsenic levels (204–412 mg/kg) and lead levels (1030–1811 mg/kg) far exceed the limits set by all reviewed international standards, including Peru (50 mg/kg As and 70–140 mg/kg Pb) [86], Canada (12 mg/kg As and 70–140 mg/kg Pb) [87], the UE (300 mg/kg Pb) [88], and the Netherlands (intervention values: 55 mg/kg As and 530 mg/kg Pb), indicating severe contamination that precludes any agricultural, residential, or even industrial use without prior remediation. Cadmium (9–16 mg/kg) exceeds agricultural thresholds (1.4–3 mg/kg) and, in some cases, residential limits (10 mg/kg), being acceptable only under industrial use (limit up to 22 mg/kg). Copper concentrations (86–136 mg/kg) exceed the values for agricultural and residential use (63–100 mg/kg) but remain below the Dutch intervention threshold (190 mg/kg). In contrast, chromium (4.6–4.8 mg/kg) and nickel (4.2–5.1 mg/kg) are well below international standards, complying with all regulations for any type of land use.

Across all treatments in Table 4, soils retained the highest metal concentrations, roots accumulated more than shoots, and TF values remained <0.40, confirming a phytostabilization rather than phytoextraction pattern. The root-to-soil BCFs we obtained are generally lower than those reported for maize grown with single amendments: for Pb, our maximum BCF (0.055, PBC500CP60) is below the 0.16 recorded for a rice husk biochar + poultry manure blend [51] and well under the 0.04–0.30 range observed for maize without compost [89]; TF-Pb values (<0.07) likewise undercut the 0.26 reported for biochar + poultry manure alone [90], indicating superior immobilization by the biochar–compost combination. For Cd, the highest BCF (0.084, run 12) is comparable to the 0.10–0.12 band cited for chars rich in oxygenated groups [50] but TF-Cd peaks (0.36, run 8) sit below the 0.50 threshold often associated with effective phytoextraction, mirroring the reduction in Cd mobility noted when biochar contains abundant surface O-functionalities [91]. Our As BCFs (≤0.059) resemble those achieved with biochar alone [92], yet TF-As remained ≤ 0.028, reinforcing reports that the 500 °C char—through higher aromaticity and concomitantly greater microbial activity—favours As retention in roots while surface O-groups are diminished [93]. The porous biochar matrix and the labile-C supply from compost together create a buffered micro-habitat that enriches sulphate-reducing, phosphate-solubilizing, and exopolysaccharide-producing bacteria [94]; these consortia immobilize metals by precipitating Pb and As as sulphides [95] or metal–phosphate minerals and by entrapping Cd and Zn in biofilms, further lowering their mobility [96]. The quinone-rich surfaces of the char act as electron shuttles, accelerating microbial redox conversions such as As (V) → As (III) and Cr (VI) → Cr (III), which yield less-soluble species that are readily sorbed on the char–compost complex [97]. In contrast, Zn, Cu, and Cr displayed moderate BCFs (Zn ≤ 0.066, Cu ≤ 0.141, Cr ≤ 0.135) far below those reported for maize with high-dose single amendments (BCF_Zn 0.82; BCF_Cu 1.08; BCF_Cr 0.21) [49]; their TF maxima (Zn 0.39, Cu 0.39, Cr 0.49) occurred at the lowest tailings dose and 300 °C, underscoring that milder pyrolysis preserves oxygenated functional groups together with microbially exuded organic ligands that facilitate upward metal transport. These comparisons show that combining compost with pine-derived biochar shifts the system toward metal immobilization, particularly at 500 °C and 60% tailings, whereas milder chars (300 °C) paired with lower tailings promote limited translocation trends consistent with the literature but delivering overall safer shoot concentrations.

### 2.4. Effect of Study Parameters

Root-level patterns (Supplementary Material Figure S3–S6): For every metal except Ni, the Root BCF rises sharply when tailings are doubled from 30% to 60%, and values are always higher with 500 °C biochar than with 300 °C; the greatest jumps occur for Cu and Cd, confirming that high-temperature biochar markedly enhances below-ground retention. Shoot-level patterns: TF generally falls as tailings increase, indicating restricted movement to aerial parts at higher contamination; 300 °C biochar maintains higher TF for Cd, Pb, Ni, and Cu, whereas 500 °C favours As (only at 30%) and Cr (at 60%), showing that the temperature effect is metal-specific. The three-dimensional plots corroborate the 2D trends of BCF maxima for the As, Cd, Ni, and Cu cluster at the extreme combination of 500 °C + 60% tailings, while Pb and Cr respond more moderately, suggesting distinct sorption/complexation behaviour. Translocation surfaces: TFs for As, Cd, Ni, and Cu decline steadily with both factors, Pb shows only a slight drop, and Cr remains low overall; collectively, these surfaces confirm that elevated pyrolysis temperature combined with higher tailings load consistently shifts metal distribution toward the root compartment.

### 2.5. Factor Model Analysis

According to the ANOVA results presented in Table 5, the BCF model for As is highly significant (F = 27.74, *p* < 0.0001). Both the tailings dose (*p* = 0.0003) and the pyrolysis temperature (*p* = 0.046), as well as their interaction (*p* = 0.0002), significantly affect arsenic accumulation in maize roots. Similarly, Cd shows an even stronger response (F = 82.12, *p* < 0.0001), where all factors—tailings dose, pyrolysis temperature, and the interaction—are highly significant (all *p* < 0.0001), underscoring Cd’s heightened sensitivity to changes in both pyrolysis conditions and contamination levels. For Pb, the overall model is also significant (F = 21.93, *p* = 0.0003). Tailings dose (*p* = 0.0002), pyrolysis temperature (*p* = 0.0029), and their interaction (*p* = 0.0387) all contribute notably, although the effect magnitudes are somewhat lower than for Cd. In contrast, Cr does not exhibit any statistically significant effect (F = 1.56, *p* = 0.2741), indicating that within the tested ranges, neither the proportion of tailings nor the pyrolysis temperature (nor their synergy) appreciably alters Cr accumulation in roots. Ni’s model is significant (F = 4.41, *p* = 0.0415). Here, tailings dose (*p* = 0.0422) and the interaction term (*p* = 0.0323) emerge as key drivers of Ni accumulation, while pyrolysis temperature alone is not significant (*p* = 0.2684). These findings show that As, Cd, Pb, and Ni accumulation in maize roots is each influenced to varying degrees by the tailings dose and pyrolysis temperature, whereas Cr appears largely unaffected under the conditions evaluated.

Table 6 clarifies which factor effects are statistically meaningful (α = 0.05). For As, the overall TF model is highly significant (F = 22.36, *p* = 0.0003); both tailings dose (*p* = 0.0003) and its interaction with pyrolysis temperature (*p* = 0.0037) contribute, whereas temperature alone is non-significant (*p* = 0.377). Cd likewise shows a significant model (F = 21.18, *p* = 0.0004), with independent main-factor effects from dose (*p* = 0.0003) and temperature (*p* = 0.001), but a non-significant interaction (*p* = 0.249), indicating additive rather than synergistic control. Pb follows a similar pattern: significant model (F = 24.35, *p* = 0.0002), significant main factors (dose *p* = 0.0001; temperature *p* = 0.002), and an interaction that trends toward but does not reach significance (*p* = 0.063). By contrast, Cr shows no significant model or factor effects (F = 0.60, *p* = 0.654), and Ni is marginal overall (F = 2.95, *p* = 0.098) with only a weak dose effect (*p* = 0.037). Cu exhibits a clearly significant model (F = 13.72, *p* = 0.0016); both dose (*p* = 0.0004) and temperature (*p* = 0.0052) act independently, while their interaction remains non-significant (*p* = 0.141). These statistics confirm that metal translocation is chiefly governed by tailings dose across most elements, augmented by pyrolysis temperature for Cd, Pb, and Cu, with synergistic dose-temperature effects evident only for As.

Based on the statistical indicators in Table 7, the Root Bioconcentration Factor (BCF) and Translocation Factor (TF) models for As, Cd, Pb, and Cu exhibit robust fits, as evidenced by high R^2^ values (≥0.84), closely aligned adjusted and predicted R^2^ values, and Adeq Precision well above 4, indicating strong explanatory and predictive capabilities. Arsenic (As) presents R^2^ values of 0.91 (BCF) and 0.89 (TF), while Cd’s BCF model stands out with an R^2^ of 0.97 and a predicted R^2^ of 0.93. Lead (Pb) shows values near 0.90 for both BCF and TF, though with slightly higher C.V. (above 27%), implying somewhat greater variability. Copper (Cu) also achieves excellent results (R^2^ = 0.97 for BCF, R^2^ = 0.84 for TF), confirming that the tested factors of biochar pyrolysis temperature and tailings dose effectively explain both root accumulation and shoot translocation for these metals. In contrast, chromium (Cr) and nickel (Ni) display weaker or even negative adjusted R^2^ values, along with substantially lower predicted R^2^, suggesting that the chosen experimental ranges do not sufficiently capture the variables governing their uptake and translocation. Overall, these findings highlight the reliability of the factorial model for As, Cd, Pb, and Cu under the studied conditions, while indicating that additional parameters or an expanded experimental range may be required to better account for Cr and Ni behaviour.

Because the aerial tissues accumulated up to 0.39 mg kg^−1^ Cd and 0.065 mg kg^−1^ Pb, direct use as fodder or food is not advisable. Two end-of-life routes are therefore recommended. (i) Thermochemical valorization: Controlled slow pyrolysis (≤550 °C) converts the biomass into a secondary biochar whose ash-enriched fraction retains >90% of the sequestered metals; the resulting char can be encapsulated in asphalt or concrete, a practice already permitted under Peruvian technical guidance. (ii) Bioenergy with ash capture: Gasification or pelletized combustion yields heat/steam while concentrating metals in <5% of the original mass; the ash can be stabilized with ordinary Portland cement and disposed of in Class I landfills. Both pathways meet Basel Convention recommendations for contaminated phytomass and close the carbon loop by generating energy.

## 3. Materials and Methods

### 3.1. Mine Tailing Sampling and Physicochemical Characterization

The tailings were sampled in Quiulacocha, considering a representative sample from four points, as shown in Figure S7, and taking into consideration the procedure carried out by the Ministry of the Environment in Peru [98]. The analysis of heavy metals was conducted after drying the mining tailings at 60 °C for 24 h [99]. The total heavy metal analysis was carried out using the ICP Aqua Regia Digestion technique.

### 3.2. Biochar and Compost Production

For the production of biochar, pine forest residues from the Cutervo district at the province of Cajamarca in Peru were obtained (6°22′31.19″ S, 78°48′6.90″ WO), as shown in Figure S1. Forest residues of pine were cut and then dried in an oven at 105 °C for 24 h [100]. The pine biochar was produced at two temperatures (300 °C and 500 °C) for 1.5 h. The biochar was named PBC300 and PBC500, and they were produced in a stainless-steel pyrolytic oven at 15 °C/min, as shown in Figure S8. The oven had a capacity of 5 L, and it had two appropriate circle endings (b) with the aim to dissipate heat. This oven also had a manometer that could register pressure from 0 to 7 bar (0–100 psi) (c), and it had a thermometer that could register an adequate temperature. Furthermore, there was a valve to regulate the gas outlet (e). Biochar yield was determined by Equation (1). The compost was produced in composting piles for three months, and the biomass to produce compost was from organic waste, such as fruits and pruning wastes from parks. It was made at the Municipality of Pueblo Libre in the district of Pueblo Libre (12°04′36.83″ S, 77°03′24.61″ W), in the province of Lima, Peru.

(1) $$Yield\;(\%)=\frac{W_{Final}}{W_{initial}}\times 100$$

where W_final_ = biomass (kg) of the pine biochar after the pyrolysis; W_initial_ = biomass (kg) of pine before the pyrolysis. Biochar and compost were granulated through mechanical sieving using a U.S.A. Standard Testing Sieve (ASTM No. 20, 850 µm) to achieve a uniform particle size distribution suitable for subsequent application (Figure S9).

### 3.3. Biochar and Compost Physicochemical Characterization

For PBC300, PBC500, and compost, pH and electrical conductivity (E.C.) (dS/m) were assessed with the methodology of Rajkovich [101] using a multiparameter (Multiparameter HANNA HI2020-02). To determine MgO and CaO, an atomic absorption spectrophotometer was used (BIOBASE-BK-AA320N) [102].

To evaluate heavy metals, such as As, Cd, Cr, Cu, Pb, Hg, Ni, and Zn (mg/kg), we used an ICP—mass spectrometry (THERMO SCIENTIFIC XSERIES 2 ICPMS), and the results were compared with international regulations. With respect to total organic matter (TOM), samples were dried at 105 °C for 10 h in desiccator, then burned at 550 °C for 6 h in muffle, and the total organic matter content was gained by the weight difference with the content of ash [103].

The elemental composition of PBC300 and PB500 (C (%), H (%), O (%) N (%), S (%)) followed the reference method of ASTM D5373 [104], using a carbon analyzer (CHN628). FTIR infrared spectroscopy analysis was performed to determine the impact of pyrolytic temperature on the conversion of functional groups on the surface of PBC300 and PBC500, for which an infrared spectrophotometer was used (Perkin Elmer Spectrum 10, wavenumber range 380 cm^−1^ to 4000 cm^−1^) [105].

In addition, in compost, total carbon (TC) was determined using the ASTM D5373 reference method [104]. Total nitrogen (TN) content was determined as follows: 1 g samples were dissolved in a certain amount of distilled water in addition to 1.2 g, 0.4 mL (1 M), and 5 mL 98%, and the total nitrogen content was measured using the Kjeldahl method [106]. Calcium and magnesium were analyzed by shaking 1 g of compost sample with sodium acetate solution for 1 h prior to atomic absorption spectroscopy detection (Varian Spectra AA 220FS, USA). The ${{N-NH}_{4}}^{+}$ and N- ${{NO}_{3}}^{-}$ amounts were extracted with 2 M of KCl [107] and determined by the AA3 Continuous Flow Analytical System. All analyses were performed in triplicate (n = 3), and the standard error (SE) was calculated.

### 3.4. Operation of the Phytoremediation System

For the ex situ phytoremediation design, four treatments were evaluated, as shown in Table 8. Each pot contained a total of 3 kg of substrate, with 3% *w*/*w* of biochar and 1.8% *w*/*w* of compost added to all treatments. This mixture was prepared uniformly for all four treatments with a tailings dose of 30% *w*/*w* and 60% *w*/*w* [108], while the remaining portion consisted of uncontaminated soil. Each treatment was coded as shown in Table 8. Direct seeding was performed with three seeds of *Zea mays* L. per experimental unit. Soil moisture conditions were maintained, as the plant requires appropriate water levels. Therefore, distilled water was used, and irrigation was applied continuously and consistently every two days [109] with an equal volume for all treatments until sample collection for laboratory analysis. Irrigation in each experimental unit was always carried out at field capacity, preventing the generation of leachates.

### 3.5. Experimental Design

A 2 × 2 factorial design was used (Table 8), in which two main factors were combined: the mining tailings dose (30% *w*/*w* and 60% *w*/*w*) and the pyrolysis temperature of pine biochar (300 °C and 500 °C). Each combination of these two factors was replicated three times, resulting in a total of twelve experimental runs, conducted in a randomized order. For each treatment, compost was incorporated, and maize (*Zea mays* L.) was cultivated. The response variables measured were the BCF and the TF for different metals.

### 3.6. Bioconcentration and Translocation Factors

The calculation of the Bioconcentration Factor (BCF) was used to assess the phystabilization of *Zea mays* L. The equation used for BCF calculation has been previously documented [110], and it is as follows:

(2) $$BCF=\frac{Concentration\;of\;metal\;in\;plant\;root}{Concentration\;of\;metal\;in\;soil}$$

The assessment of the Translocation Factor (TF) plays a crucial role in assessing the metabolic functions and well-being of individuals plants that thrive in polluted environments [111]. TF was determined by the ratio of metal concentration in plant aerial parts (shoot) and metal concentration in plant roots [112], and the equation was the following:

(3) $$TF=\frac{Metal\;concentration\;in\;plant\;shoot}{Metal\;concentration\;in\;plant\;root}$$

### 3.7. Statistical Analysis

For this research, a factorial design was developed as a statistical regression tool. This method allows for a mathematical relationship between the response variable (Y) and the independent variables (X_1_, X_2_, X_3_), as shown in the following equation:

(4) $$Y=\beta_{0}+\beta_{1}X_{1}{+\beta }_{2}X_{2}{+\beta }_{3}X_{3}{+\beta }_{12}{X_{1}X}_{2}\beta_{13}{X_{1}X}_{3}{+\beta }_{23}{X_{2}X}_{3}{+\beta }_{123}{{X_{1}X}_{2}X}_{3}$$

where Y is the predicted response used as the dependent variable; X_i_ (*i* = 1, 2, and 3) represents the parameters; β_0_ can be calculated by dividing the system responses (Y) by the total number of observations, including those conducted at the central point; and β_i_ (*i* = 1, 2, and 3) and β_ij_ (*i* = 1, 2, and 3; *j* = 1, 2, and 3) represent the effect coefficients [1].

Subsequently, an analysis of variance (ANOVA) was conducted to evaluate the quality and robustness of the fitted models for each response variable. The R^2^ (coefficient of determination) represents the proportion of variation explained by the model, while the adjusted R^2^ corrects for the number of model terms to prevent overestimation. The predicted R^2^ assesses the model’s generalization ability by indicating how much variability is explained when applied to new data. Adeq Precision (Adequate Precision) measures the signal-to-noise ratio, with values above 4 considered sufficient for distinguishing real effects from experimental noise. The standard deviation (Std. Dev.) quantifies residual dispersion (differences between observed and predicted values), while the mean helps interpret this dispersion relative to the average value. Lastly, the coefficient of variation (C.V.%) relates the standard deviation to the mean, serving as an indicator of whether residual variability is high or low in relative terms.

## 4. Conclusions

The results of this study demonstrate that the combined application of biochar and compost significantly enhances the remediation of mine tailing-contaminated soils. This factorial study demonstrates that co-amending mine tailing-contaminated soil with 3% w/w pine-derived biochar and 1.8% *w*/*w* compost markedly improves substrate pH, cation exchange capacity, and nutrient retention while reducing the bioavailability and shoot translocation of priority metals (As, Cd, Pb, Cu, Cr, Ni). The combination of the higher tailings load (60%) with the hotter char (500 °C) achieved the greatest immobilization, keeping Translocation Factors below 0.07 for Pb and <0.03 for As. Future work will (i) upscale the study to field conditions, first establishing maize and then an additional short-cycle food crop to quantify yields and whole-plant metal uptake under ambient climate and irrigation; (ii) incorporate BET surface area determinations and test chars produced at ≥600 °C to assess whether any extra porosity gained offsets the concurrent loss of surface functional groups; (iii) benchmark maize against regionally native hyper-accumulator species, alone and in mixed-cropping schemes, to identify synergistic combinations; (iv) track metal speciation, soil enzymatic activity, and rhizosphere microbiome composition across successive cropping cycles to verify the long-term chemical and biological stability of the remediation strategy; and (v) translate the findings into policy by providing baseline thresholds and amendment-rate guidelines that can inform national soil quality standards and regional land use regulations, thereby supporting evidence-based decisions for the safe rehabilitation and productive reuse of mine-affected soils.

## Figures and Tables

**Table 1:** Physicochemical characterization of the Quiulacocha mining tailings.

Heavy Metals in Mining Tailings						
As (mg/kg)	Cd (mg/kg)	Cr (mg/kg)	Cu (mg/kg)	Ni (mg/kg)	Pb (mg/kg)	Zn (mg/kg)
1015.33 ± 5.03	17 ± 1.00	40.67 ± 0.58	288.30 ± 1.57	9.60 ± 0.53	3983.67 ± 29.50	9091.00 ± 79.96
**Physicochemical Characteristics of Mining Tailings**						
pH	EC	CaCO_3_ (%)	OM (%)	P (ppm)	K (ppm)	C.E.C. (meq/100 g)
5.63 ± 0.02	7.14 ± 0.02	0.35 ± 0.01	3.02 ± 0.01	0.82 ± 0.03	96 ± 1.00	4.33 ± 0.02

**Table 2:** Physicochemical characterization of biochar and compost.

Parameter	PB300	PB500	Compost
C (%)	72.27 ± 1.00	80.870 ± 1.589	20.92 ±1.12
H (%)	4.22 ± 0.07	3.357 ± 0.124	-
O (%)	24.22 ± 1.00	17.887 ± 1.169	-
N (%)	0.460 ± 0.03	0.603 ± 0.025	1.79 ± 0.10
S (%)	0.017 ± 0.003	0.042 ± 0.006	-
E.C. (uS/cm)	169.433 ± 5.164	255.60 ± 10.923	11.29 ± 1.35
TOM (%)	54.597 ± 1.511	39.940 ± 2.359	35.02 ± 0.97
pH	7.133 ± 0.071	7.933 ± 0.012	8.53 ± 0.25
CaO (%)	0.147 ± 0.006	0.132 ±0.007	3.60 ± 0.49
MgO (%)	0.052 ± 0.011	0.136 ± 0.038	0.64 ± 0.06
H/C	0.058	0.041	-
O/C	0.33	0.22	-
NO_3_	-	-	13.55 ± 1.18
NH_4_	-	-	1.35 ± 0.07

**Table 3:** Heavy metal characterization of biochar and compost with different international regulations.

Heavy Metal	Biochar						Compost			
	IBI	EBC	G	AU	PBC300	PBC500	Korea	EU	USA	Compost
As (mg/kg)	≤100	<13	≤40	≤40	13.46 ± 0.772	24.453 ± 0.086	45	25	41	14.34 ± 0.48
Cd (mg/kg)	≤39	<1.5	≤1.5	≤3	<0.0001 ± 0.00	<0.0001 ± 0.00	5	0.7–10	39	2.23 ± 0.26
Cr (mg/kg)	≤1200	<90	/	/	<0.0003 ± 0.00	<0.0003 ± 0.00	200	70–200	1200	0.00021 ± 0.00
Cu (mg/kg)	≤6000	<100	/	/	0.0002 ± 0.00	0.8733 ± 0.086	360	70–600	1500	54.94 ± 2.32
Pb (mg/kg)	300	<150	≤150	≤100	0.0020 ± 0.001	0.0020 ± 0.000	130	70–1000	300	28.44 ± 2.31
Ni (mg/kg)	≤420	<50	≤80	≤100	<0.0003 ± 0.00	<0.0003 ± 0.00	45	20–200	420	0.00026 ± 0.00
Zn (mg/kg)	≤7400	<400	/	/	0.0001 ± 0.00	23.30 ± 3.176	900	210–4000	2800	173.63 ± 3.46

**Table 4:** Experimental results of BFC and TF for As, Cd, Pb, Cr, Ni, and Cu in corn (*Zea mays* L.), under different tailings doses and pyrolysis temperatures.

Run	B: Pyrolysis Temperature °C	A: Doses Mining Tailing % (*w*/*w*)	FBC-As	FT-As	FBC-Cd	FT-Cd	FBC-Pb	FT-Pb	FBC-Cr	FT-Cr	FBC-Ni	FT-Ni	FBC-Cu	FT-Cu
1	500	30	0.012	0.023	0.018	0.182	0.015	0.011	0.101	0.208	0.043	0.217	0.035	0.111
2	500	60	0.059	0.004	0.068	0.043	0.037	0.006	0.100	0.222	0.070	0.147	0.122	0.018
3	300	60	0.015	0.011	0.019	0.229	0.022	0.011	0.109	0.260	0.074	0.171	0.066	0.079
4	300	30	0.039	0.022	0.013	0.308	0.005	0.040	0.066	0.313	0.047	0.250	0.014	0.290
5	300	60	0.015	0.010	0.024	0.154	0.029	0.010	0.090	0.239	0.072	0.167	0.066	0.067
6	500	30	0.008	0.037	0.018	0.316	0.011	0.029	0.087	0.225	0.048	0.316	0.053	0.067
7	500	30	0.010	0.028	0.030	0.190	0.012	0.017	0.077	0.314	0.048	0.300	0.055	0.198
8	300	30	0.023	0.017	0.015	0.364	0.004	0.039	0.113	0.240	0.071	0.267	0.019	0.224
9	300	60	0.018	0.012	0.024	0.212	0.020	0.013	0.058	0.385	0.038	0.294	0.053	0.125
10	300	30	0.023	0.015	0.013	0.308	0.004	0.034	0.097	0.488	0.071	0.400	0.019	0.394
11	500	60	0.045	0.004	0.077	0.030	0.064	0.006	0.135	0.318	0.112	0.164	0.124	0.017
12	500	60	0.049	0.004	0.084	0.035	0.064	0.003	0.119	0.400	0.099	0.200	0.141	0.024

**Table 5:** ANOVA for BCF of As, Cd, Pb, Cr, Ni, and Cu in *Zea mays* L. under different mining tailings doses and pyrolysis temperatures.

Source	Sum of Squares	df	Mean Square	F-Value	*p*-Value
BFC-As = 0.026 + 0.007 × A + 0.004 × B +0.014 × AB					
Model	0.003	3	0.001	27.74	0.0001
A-Mine Tailings Dosage	0.0006	1	0.0006	16.83	0.0034
B-Pyrolysis Temperature	0.0002	1	0.0002	5.57	0.046
AB	0.0022	1	0.0022	60.8	<0.0001
Pure Error	0.0003	8	0		
Cor Total	0.0033	11			
BFC-Cd = 0.034 + 0.0159 × A + 0.0157 × B + 0.012 × AB					
Model	0.0075	3	0.0025	82.12	<0.0001
A-Mine Tailings Dosage	0.003	1	0.003	98.59	<0.0001
B-Pyrolysis Temperature	0.003	1	0.003	96.69	<0.0001
AB	0.0016	1	0.0016	51.09	<0.0001
Pure Error	0.0002	8	0		
Cor Total	0.0078	11			
BFC-Pb = 0.024 + 0.015 × A + 0.009 × B + 0.006 × AB					
Model	0.0044	3	0.0015	21.99	0.0003
A-Mine Tailings Dosage	0.0028	1	0.0028	41.94	0.0002
B-Pyrolysis Temperature	0.0012	1	0.0012	17.94	0.0029
AB	0.0004	1	0.0004	6.1	0.0387
Pure Error	0.0005	8	0.0001		
Cor Total	0.0049	11			
BFC-Cr = 0.096 + 0.006 × A + 0.008 × B + 0.009 × AB					
Model	0.002	3	0.0007	1.56	0.2741
A-Mine Tailings Dosage	0.0004	1	0.0004	0.9484	0.3586
B-Pyrolysis Temperature	0.0006	1	0.0006	1.44	0.2647
AB	0.001	1	0.001	2.28	0.1695
Pure Error	0.0034	8	0.0004		
Cor Total	0.0054	11			
BFC-Ni = 0.066 + 0.0116 × A + 0.004 × B + 0.012 × AB					
Model	0.0036	3	0.0012	4.41	0.0415
A-Mine Tailings Dosage	0.0016	1	0.0016	5.83	0.0422
B-Pyrolysis Temperature	0.0002	1	0.0002	0.7126	0.4231
AB	0.0018	1	0.0018	6.68	0.0323
Pure Error	0.0022	8	0.0003		
Cor Total	0.0057	11			
BFC-Cu = 0.064 + 0.031 × A + 0.024 × B + 0.009 × AB					
Model	0.0201	3	0.0067	91.1	<0.0001
A-Mine Tailings Dosage	0.0119	1	0.0119	161.47	<0.0001
B-Pyrolysis Temperature	0.0072	1	0.0072	97.52	<0.0001
AB	0.0011	1	0.0011	14.32	0.0054
Pure Error	0.0006	8	0.0001		
Cor Total	0.0207	11			

**Table 6:** ANOVA for TF of As, Cd, Pb, Cr, Ni, and Cu in *Zea mays* L. under different tailings doses and biochar pyrolysis temperatures.

Source	Sum of Squares	df	Mean Square	F-Value	*p*-Value
TF-As = 0.015 + −0.008 × A + 0.001 × B + −0.005 × AB					
Model	0.0011	3	0.0004	22.36	0.0003
A-Mine Tailings Dosage	0.0008	1	0.0008	49.75	0.0001
B-Pyrolysis Temperature	0	1	0	0.8755	0.3768
AB	0.0003	1	0.0003	16.45	0.0037
Pure Error	0.0001	8	0		
Cor Total	0.0012	11			
TF-Cd = 0.197 + −0.080 × A + −0.06 × B + −0.016 × AB					
Model	0.131	3	0.0437	21.18	0.0004
A-Mine Tailings Dosage	0.0775	1	0.0775	37.58	0.0003
B-Pyrolysis Temperature	0.0503	1	0.0503	24.42	0.0011
AB	0.0032	1	0.0032	1.54	0.2493
Pure Error	0.0165	8	0.0021		
Cor Total	0.1475	11			
TF-Pb = 0.018 + −0.010 × A + −0.006 × B + 0.0035 × AB					
Model	0.0018	3	0.0006	24.35	0.0002
A-Mine Tailings Dosage	0.0012	1	0.0012	49.53	0.0001
B-Pyrolysis Temperature	0.0005	1	0.0005	18.86	0.0025
AB	0.0001	1	0.0001	4.67	0.0627
Pure Error	0.0002	8	0		
Cor Total	0.002	11			
TF-Cr = 0.300 + 0.003 × A + −0.019 × B + 0.029 × AB					
Model	0.0151	3	0.005	0.5957	0.6353
A-Mine Tailings Dosage	0.0001	1	0.0001	0.0131	0.9116
B-Pyrolysis Temperature	0.0047	1	0.0047	0.5574	0.4767
AB	0.0102	1	0.0102	1.22	0.3021
Pure Error	0.0674	8	0.0084		
Cor Total	0.0825	11			
TF-Ni = 0.241 + −0.050 × A + −0.017 × B + −0.003 × AB					
Model	0.0343	3	0.0114	2.95	0.0984
A-Mine Tailings Dosage	0.0307	1	0.0307	7.91	0.0227
B-Pyrolysis Temperature	0.0035	1	0.0035	0.901	0.3703
AB	0.0001	1	0.0001	0.0306	0.8655
Pure Error	0.031	8	0.0039		
Cor Total	0.0653	11			
TF-Cu = 0.135 + −0.079 × A + −0.062 × B + 0.027 × AB					
Model	0.1306	3	0.0435	13.72	0.0016
A-Mine Tailings Dosage	0.076	1	0.076	23.94	0.0012
B-Pyrolysis Temperature	0.0461	1	0.0461	14.53	0.0052
AB	0.0085	1	0.0085	2.68	0.1405
Pure Error	0.0254	8	0.0032		
Cor Total	0.156	11			

**Table 7:** Statistical indicators (ANOVA) of the factorial models of the Root Bioconcentration Factor (BCF) and the Translocation Factor (TF) for As, Cd, Pb, Cr, Ni, and Cu.

Model Indicators	BFC-As	TF-As	BFC-Cd	TF-Cd	BFC-Pb	TF-Pb	BFC-Cr	TF-Cr	BFC-Ni	TF-Ni	BFC-Cu	TF-Cu
R^2^	0.91	0.89	0.97	0.89	0.89	0.90	0.37	0.18	0.62	0.53	0.97	0.84
Adjusted R^2^	0.88	0.85	0.96	0.85	0.85	0.86	0.13	−0.12	0.48	0.35	0.96	0.78
Predicted R^2^	0.80	0.76	0.93	0.75	0.76	0.78	−0.42	−0.84	0.15	−0.07	0.94	0.63
Adeq Precision	11.90	11.11	19.76	11.07	10.71	11.38	2.71	1.85	5.00	3.76	22.58	8.70
Std. Dev.	0.01	0.00	0.01	0.05	0.01	0.00	0.02	0.09	0.02	0.06	0.01	0.06
Mean	0.03	0.02	0.03	0.20	0.02	0.02	0.10	0.30	0.07	0.24	0.06	0.13
C.V.%	22.87	25.77	16.46	22.99	34.25	27.25	21.59	30.49	24.86	25.82	13.43	41.86

**Table 8:** Factorial design.

Biochar	Compost	Mining Tailing (% *w*/*w*)	Component	Codification
PBC300	YES	30%	Soil + *Zea mays* L.	PBC300CP30
PBC300	YES	60%	Soil + *Zea mays* L.	PBC300CP60
PBC500	YES	30%	Soil + *Zea mays* L.	PBC500CP30
PBC500	YES	60%	Soil + *Zea mays* L.	PBC500CP60

## Data Availability

All the data are included in the manuscript.

## Supplementary Materials

The following supporting information can be downloaded at: https://www.mdpi.com/article/10.3390/plants14101448/s1. Figure S1. Location of the pine harvesting site and municipal composting plant. Figure S2. FTIR analysis of biochar (PBC300 (a) and PBC500 (b)). Figure S3. Interaction of Mining Tailings Dose and Pine Biochar Pyrolysis Temperature on BCF for heavy metals. Figure S4. Interaction of Mining Tailings Dose and Pine Biochar Pyrolysis Temperature on TF for heavy metals. Figure S5. 3D Response Surfaces of BCF for As, Cd, Pb, Cr, Ni, and Cu as a Function of Biochar Pyrolysis Temperature and Mining Tailings Dose. Figure S6. 3D Response Surfaces of TF for As, Cd, Pb, Cr, Ni, and Cu as a Function of Biochar Pyrolysis Temperature and Mining Tailings Dose. Figure S7. Location of sampling points at the Quiulacocha mining tailings in Pasco. Figure S8. Compost and biochar production. Figure S9. Granulation of biochar and compost using ASTM No. 20 sieve. Table S1. BCF and TF of the treatments.
