# Supplementary material for: Integrated Biochar–Compost Amendment for Zea mays L. Phytoremediation in Soils Contaminated with Mining Tailings of Quiulacocha, Peru
Source: Plants (Basel). 2025 May 12;14(10):1448. doi: 10.3390/plants14101448 (PMC12115018; doi:10.3390/plants14101448)
Supplement: Supplementary file 1 [file plants-14-01448-s001.zip › plants-3540804-supplementary.pdf]

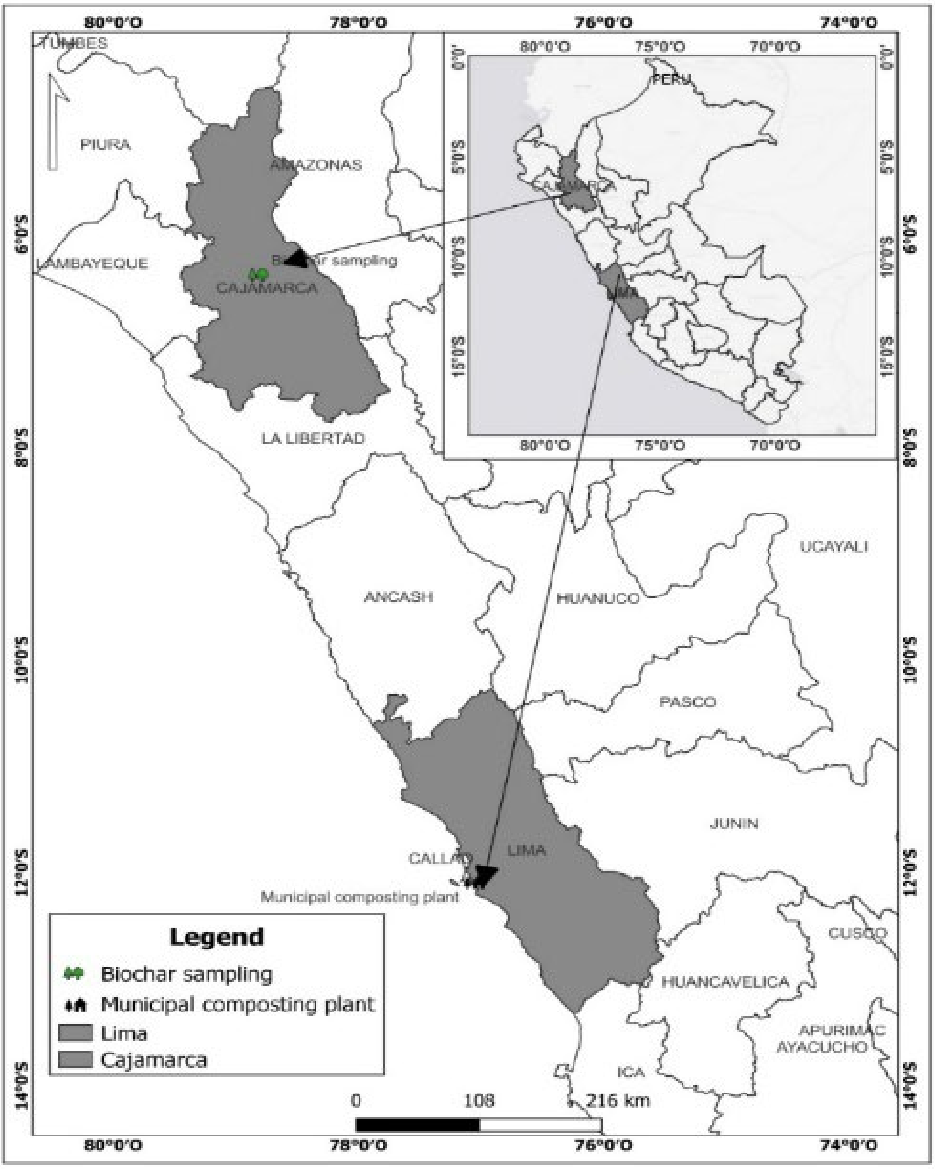

Figure S1. Location of the pine harvesting site and municipal composting plant

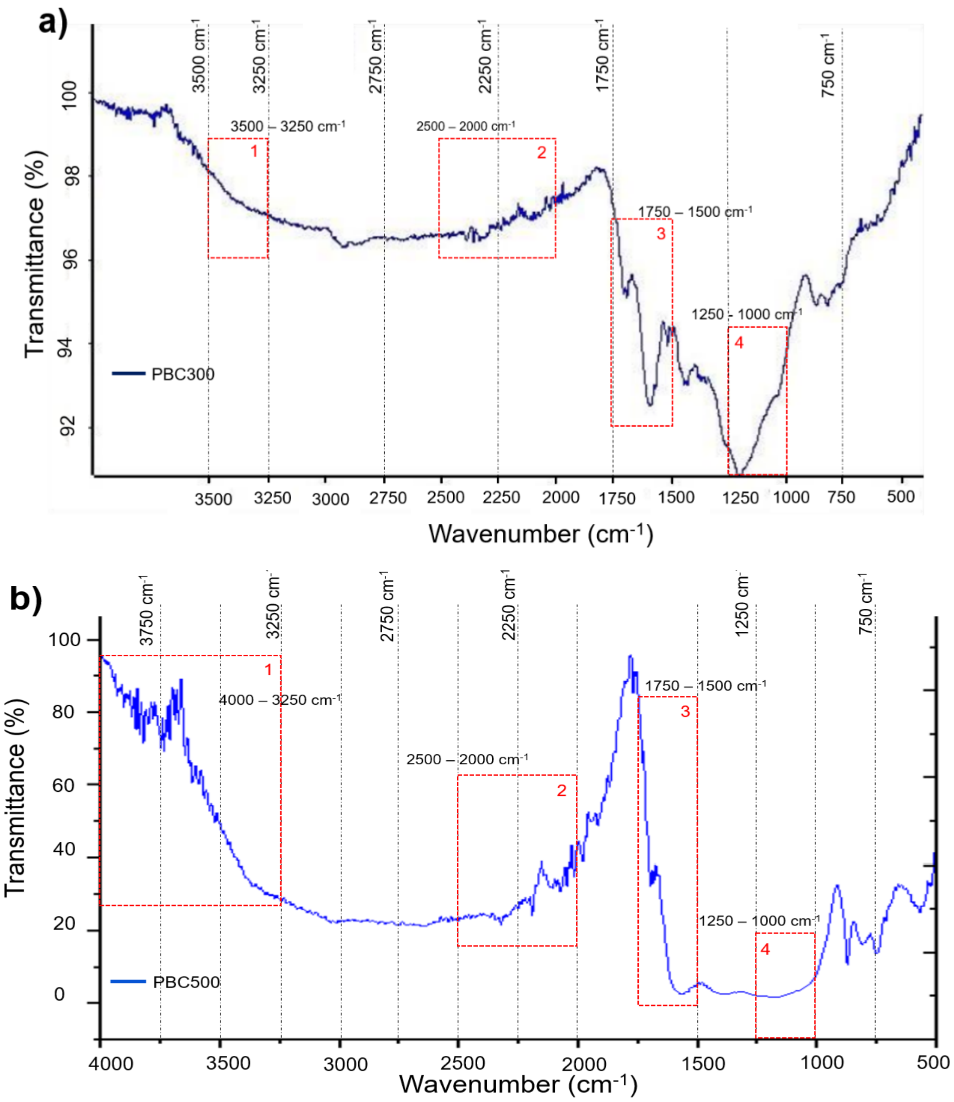

Figure S2. FTIR analysis of biochar (PBC300 (a) and PBC500 (b)).

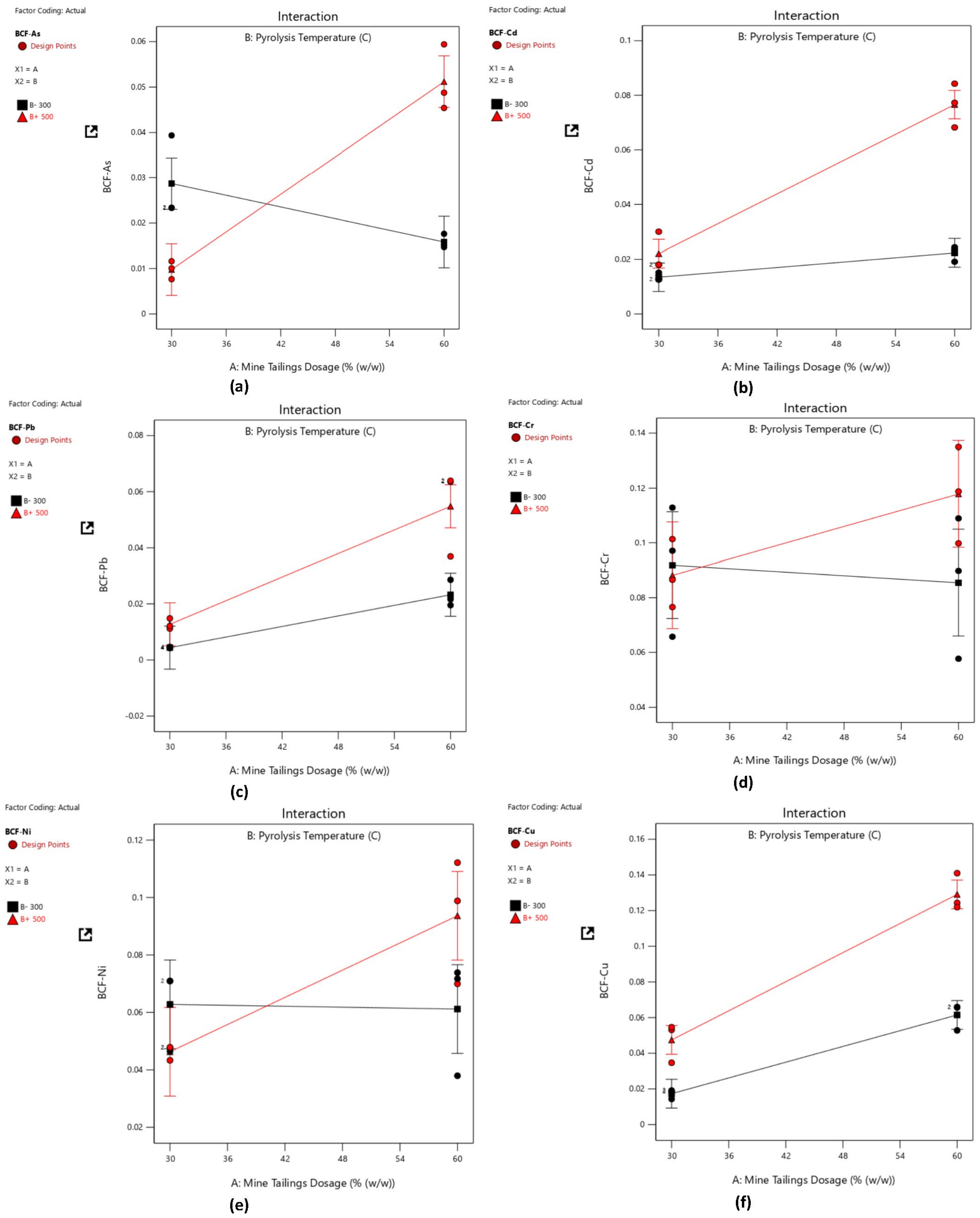

Figure S3. Interaction of Mining Tailings Dose and Pine Biochar Pyrolysis Temperature on BCF for heavy metals.

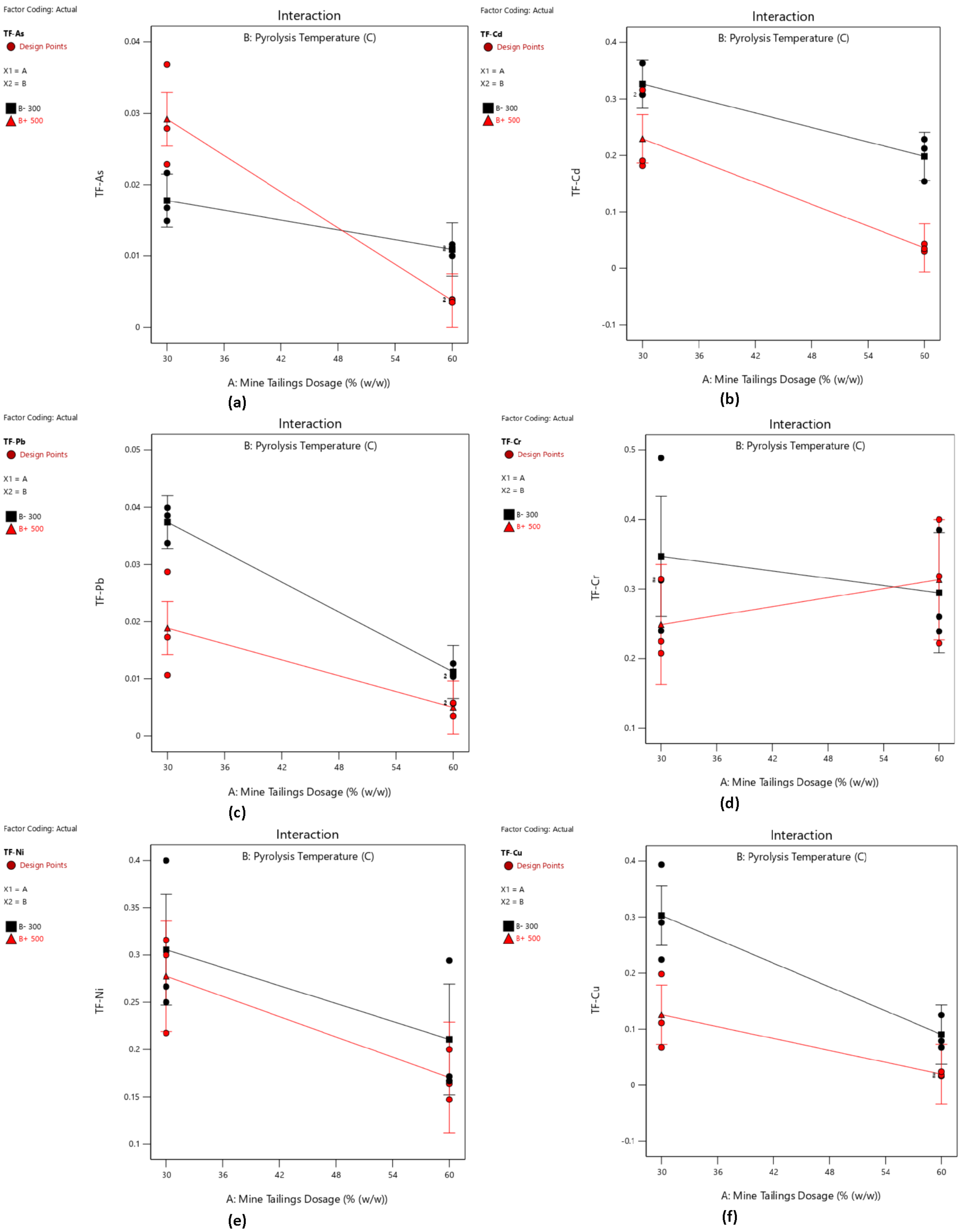

Figure S4. Interaction of Mining Tailings Dose and Pine Biochar Pyrolysis Temperature on TF for heavy metals.

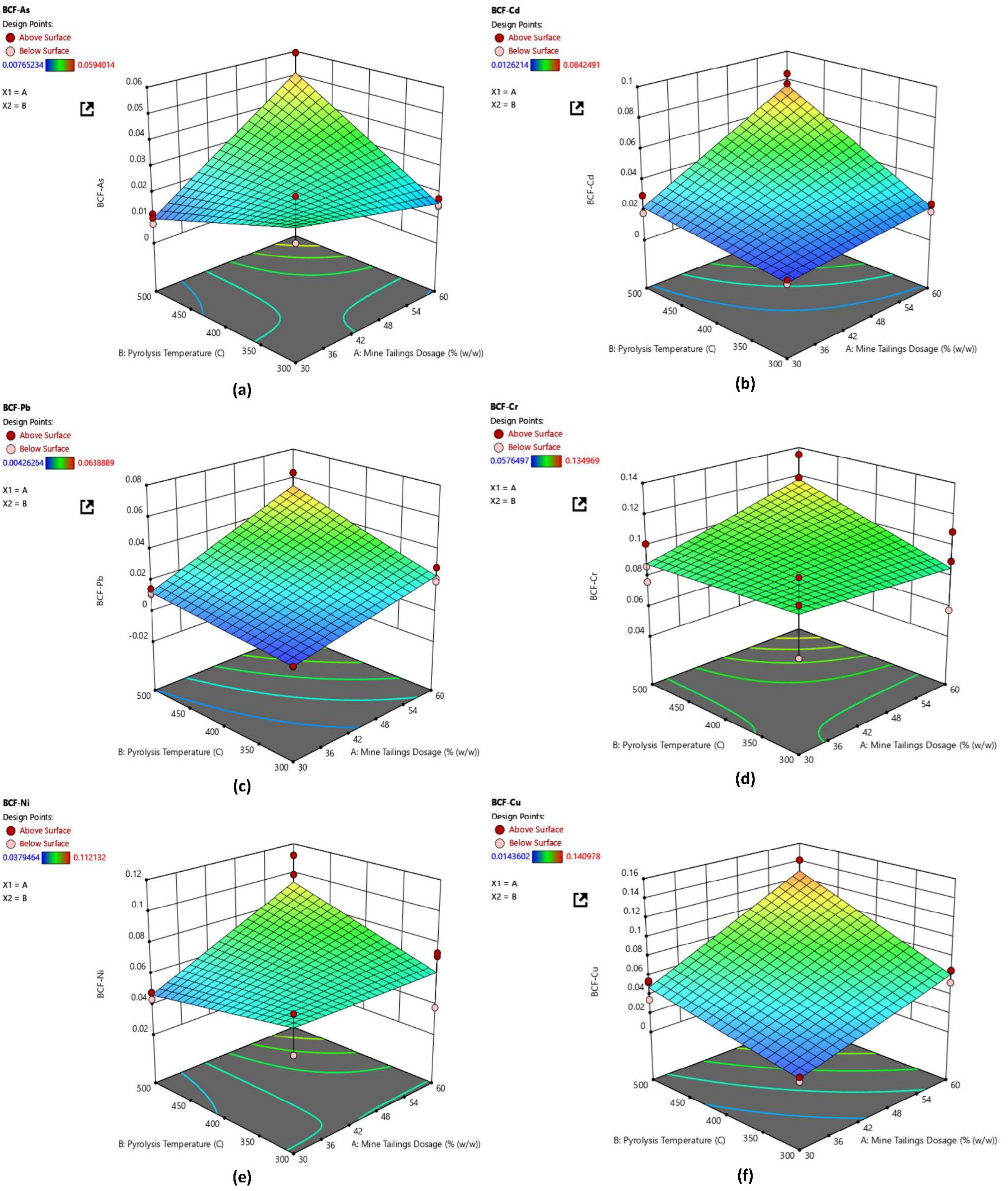

Figure S5. 3D Response Surfaces of BCF for As, Cd, Pb, Cr, Ni, and Cu as a Function of Biochar Pyrolysis Temperature and Mining Tailings Dose.

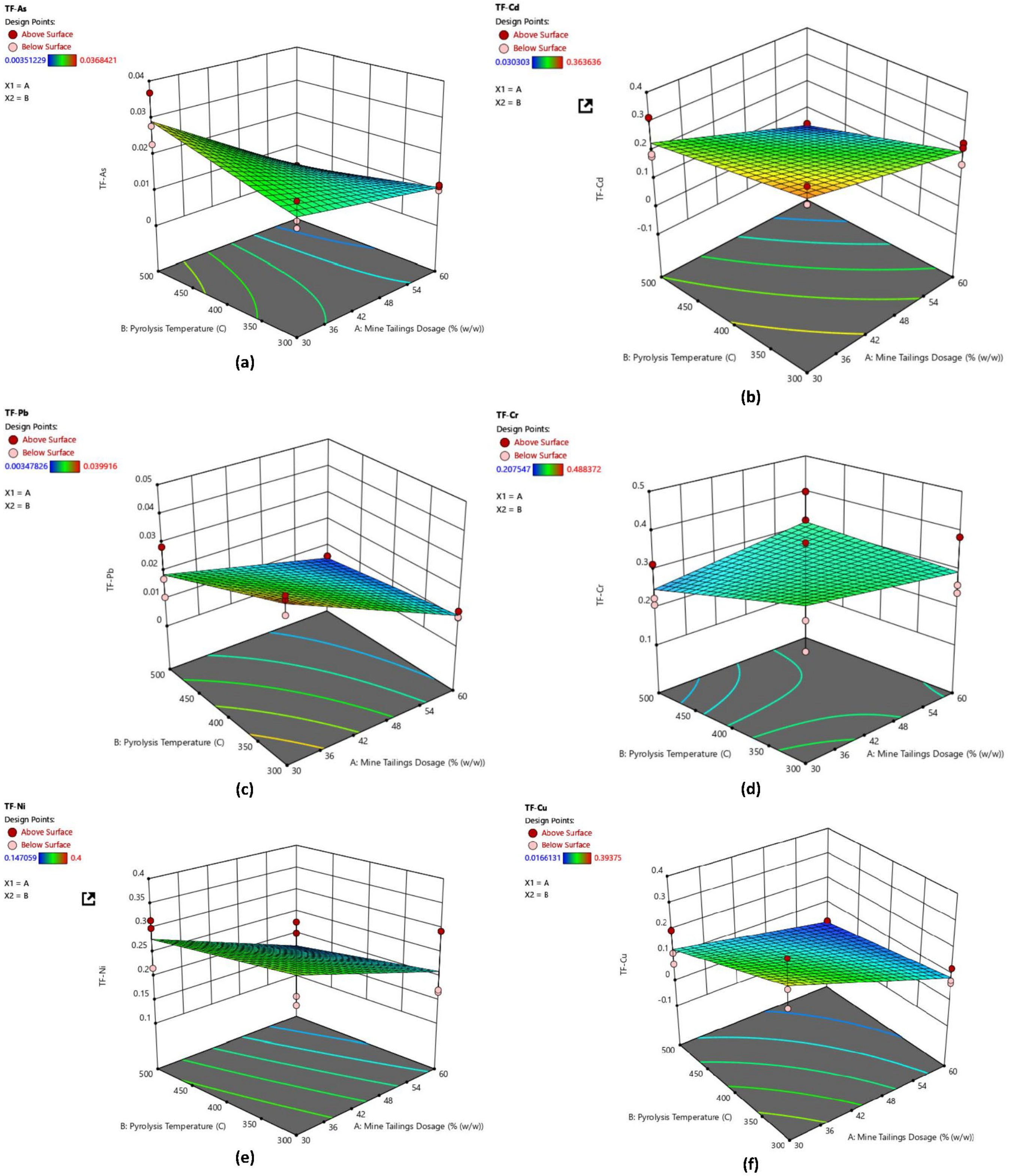

Figure S6. 3D Response Surfaces of TF for As, Cd, Pb, Cr, Ni, and Cu as a Function of Biochar Pyrolysis Temperature and Mining Tailings Dose.

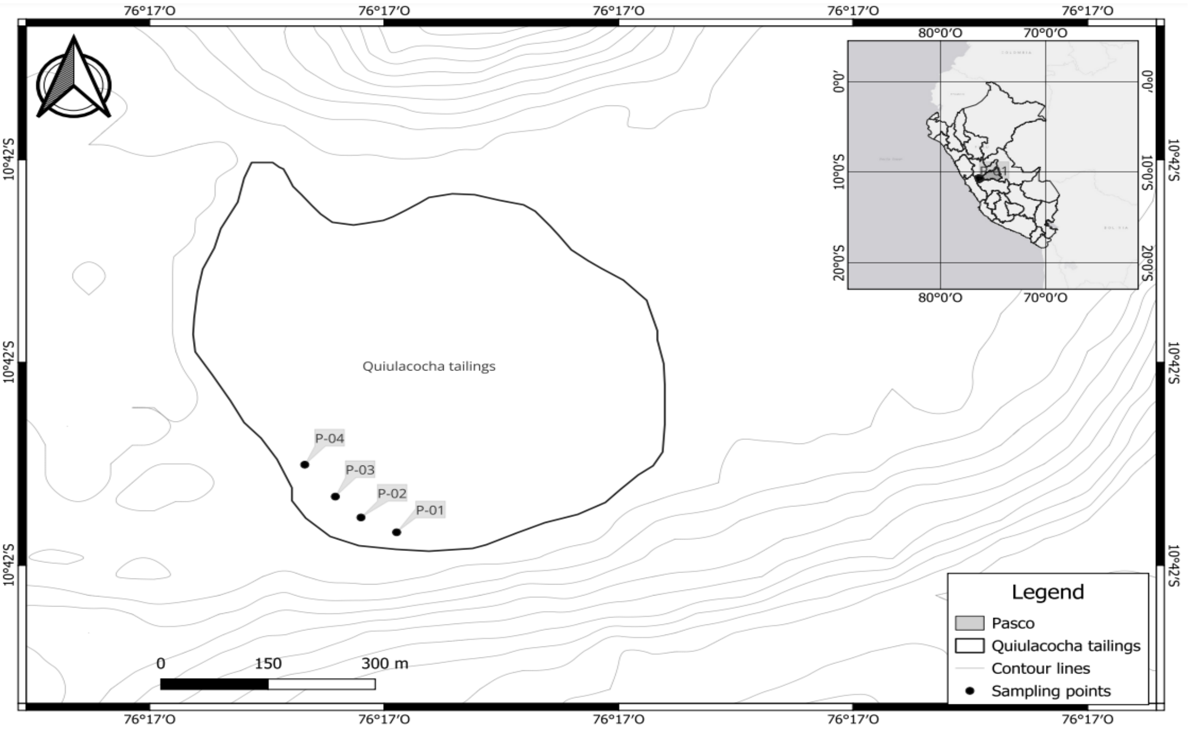

Figure S7. Location of sampling points at the Quiulacocha mining tailings in Pasco.

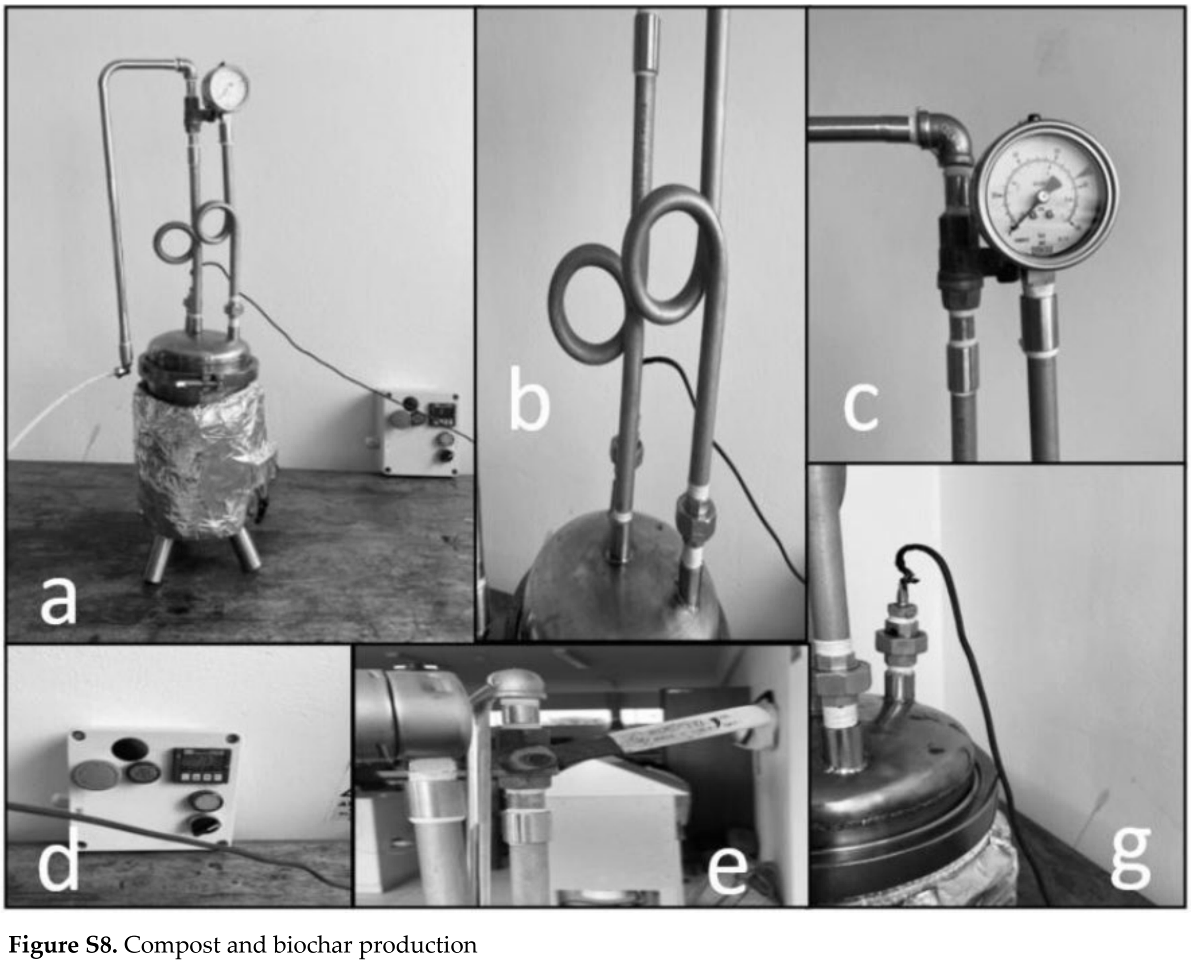

Figure S8. Compost and biochar production

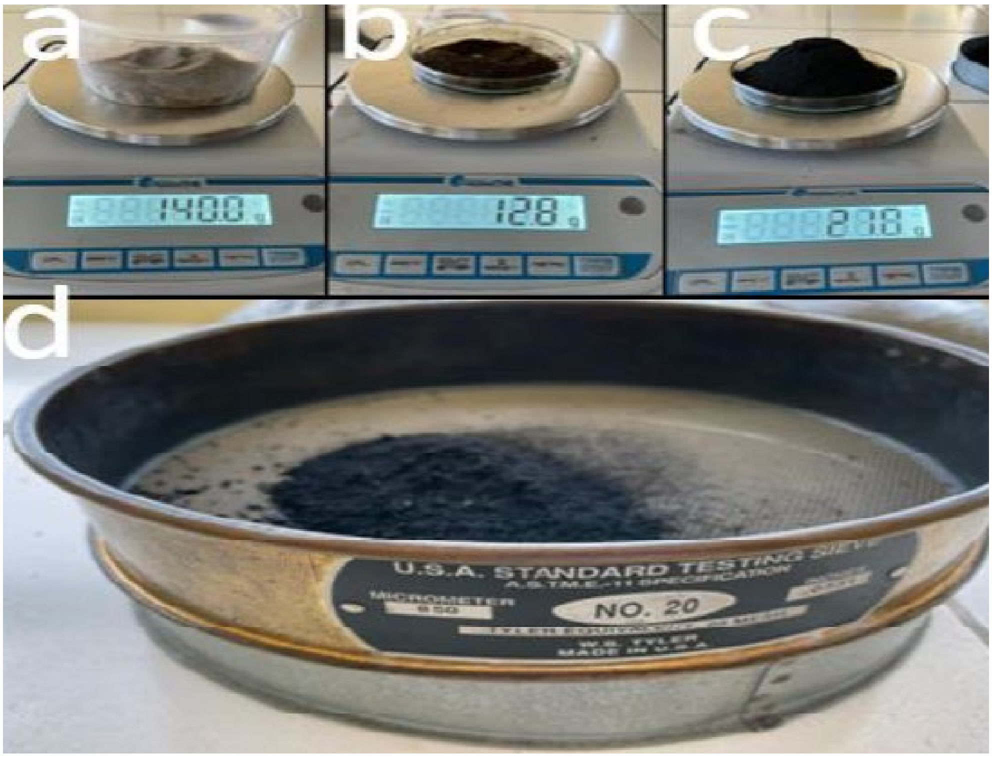

Figure S9. Granulation of biochar and compost using ASTM No. 20 sieve

Table S1. BCF and TF of the treatments

Treatment	Component	As (mg/kg)	Cd (mg/kg)	Cr (mg/kg)	Cu (mg/kg)	Pb (mg/kg)	Ni (mg/kg)
PBC300CP30	root	5.58 ± 0.36	0.12 ± 0.01	0.42 ± 0.09	1.49 ± 0.22	4.54 ± 0.19	0.27 ± 0.06
	leaves	0.1 ± 0.02	0.04 ± 0.00	0.14 ± 0.06	0.45 ± 0.15	0.17 ± 0.02	0.083 ± 0.04
	soil	204.27 ± 44.90	9.29 ± 1.76	4.58 ± 0.25	86.35 ± 0.04	1031.65 ± 5.78	4.25 ± 0.04
	TF	1.78E-02 ± 3.50E-03	3.26E-01 ± 3.23E-02	3.47E-01 ± 1.28E-01	3.03E-01 ± 8.54E-02	3.74E-02 ± 3.26E-03	3.06E-01 ± 8.22E-02
	BCF	2.87E-02 ± 9.26E-03	1.35E-02 ± 1.46E-03	9.19E-02 ± 2.40E-02	1.73E-02 ± 2.59E-03	4.40E-03 ± 2.11E-04	6.28E-02 ± 1.41E-02
PBC300CP60	root	6.45 ± 0.49	0.47 ± 0.19	0.41 ± 0.13	7.96 ± 2.12	42.88 ± 11.40	0.29 ± 0.11
	leaves	0.07 ± 0.00	0.07 ± 0.01	0.11 ± 0.02	0.67 ± 0.03	0.47 ± 0.09	0.057 ± 0.01
	soil	411.77 ± 67.03	16.13 ± 2.39	4.74 ± 0.33	127.97 ± 20.72	1831 ± 109.33	4.75 ± 0.27
	TF	1.09E-02 ± 8.13E-04	1.98E-01 ± 3.93E-02	2.95E-01 ± 7.87E-02	9.04E-02 ± 3.07E-02	1.12E-02 ± 1.28E-03	2.11E-01 ± 7.22E-02
	BCF	1.58E-02 ± 1.58E-03	2.23E-02 ± 2.80E-03	8.55E-02 ± 2.59E-02	6.15E-02 ± 7.50E-03	2.32E-02 ± 4.74E-03	6.12E-02 ± 2.01E-02
PBC500CP30	root	2.49 ± 0.58	0.21 ± 0.02	0.43 ± 0.09	4.25 ± 0.64	14.15 ± 2.47	0.21 ± 0.02
	leaves	0.07 ± 0.00	0.045 ± 0.01	0.10 ± 0.01	0.54 ± 0.33	0.25 ± 0.09	0.057 ± 0.01
	soil	254.13 ± 8.68	9.24 ± 3.77	4.81 ± 0.37	90.92 ± 8.93	1110.51 ± 27.64	4.49 ± 0.72
	TF	2.92E-02 ± 7.08E-03	2.29E-01 ± 7.50E-02	2.49E-01 ± 5.73E-02	1.26E-01 ± 6.66E-02	1.89E-02 ± 9.13E-03	2.78E-01 ± 5.28E-02
	BCF	1.52E-02 ± 9.88E-03	2.20E-02 ± 6.90E-03	8.82E-02 ± 1.25E-02	4.75E-02 ± 1.11E-02	1.27E-02 ± 1.89E-03	4.63E-02 ± 2.59E-03
PBC500CP60	root	18.73 ± 1.05	1.21 ± 0.10	0.55 ± 0.11	17.43 ± 1.08	99.03 ± 27.23	0.48 ± 0.14
	leaves	0.07 ± 0.00	0.043 ± 0.01	0.18 ± 0.07	0.34 ± 0.05	0.48 ± 0.15	0.08 ± 0.03
	soil	370.82 ± 55.20	15.92 ± 1.96	4.68 ± 0.19	135.84 ± 16.47	1810.94 ± 17.35	5.12 ± 0.29
	TF	3.74E-03 ±2.03E-04	3.61E-02 ± 6.50E-03	3.13E-01 ± 8.90E-02	1.96E-02 ± 3.93E-03	4.97E-03 ± 1.30E-03	1.70E-01 ± 2.70E-02
	BCF	5.12E-02 ± 7.30E-03	7.66E-02 ± 8.05E-03	1.18E-01 ± 1.76E-02	1.29E-01 ± 1.04E-02	5.48E-02 ± 1.55E-02	9.36E-02 ± 2.16E-02
